# Strategies to Support Physical Activity Participation in Older Adults With Heart Failure

**DOI:** 10.1093/geroni/igaa057.1469

**Published:** 2020-12-16

**Authors:** Katherine Hall, Amy Pastva, Heather King, Sean Lowers, Julie Miller, Jennie Riley

**Affiliations:** 1 Duke University, Veterans Health Administration, Durham, North Carolina, United States; 2 Duke University School of Medicine, Durham, North Carolina, United States; 3 Duke University, Durham, United States; 4 Duke Roybal Center, Durham, North Carolina, United States

## Abstract

Physical activity (PA) is recommended for people living with heart failure (HF). Despite evidence of its benefits, participation in PA is low in this population, putting them at risk for loss of functional independence and additional health burdens. The aim of this pilot study was to ask older adults living with stable, chronic HF to identify strategies to support regular PA. Patients in an outpatient HF rehabilitation program were recruited to participate in focus groups about their PA knowledge, attitudes, and preferences as part of a stakeholder engagement project. At the beginning of the focus group, participants completed a questionnaire listing 8 potential strategies to optimize PA, and were asked to identify the top 4 strategies that they thought would be most beneficial to support regular PA participation. This was the focus of the current analysis. Thirteen adults with HF (M age=65; 46% female; 62% African American; M BMI=32.6 kg/m2) were enrolled. Top-rated strategies endorsed by participants to support long-term adherence to PA included provision of an exercise guide to support home-based exercise and supplement health provider-supervised exercise sessions (69%), group education classes (64%), completion of fitness assessments at regular intervals (62%), and provision of a transition pathway from an exercise rehabilitation program to a community-based exercise program (62%). The remaining strategies were endorsed by fewer than 50% of participants, and included remote delivery and support options. These results have important implications for future program development and implementation efforts to support PA among older adults with stable, chronic HF.

